# Transcriptome profiling of interaction effects of soybean cyst nematodes and soybean aphids on soybean

**DOI:** 10.1038/s41597-019-0140-4

**Published:** 2019-07-24

**Authors:** Surendra Neupane, Febina M. Mathew, Adam J. Varenhorst, Madhav P. Nepal

**Affiliations:** 1 Department of Biology and Microbiology, South Dakota State University, Brookings, SD 57007 USA; 2 Department of Agronomy, Horticulture and Plant Science, South Dakota State University, Brookings, SD 57007 USA

**Keywords:** Biotic, Transcriptomics, RNA sequencing

## Abstract

Soybean aphid (*Aphis glycines*; SBA) and soybean cyst nematode (*Heterodera glycines*; SCN) are two major pests of soybean (*Glycine max*) in the United States of America. This study aims to characterize three-way interactions among soybean, SBA, and SCN using both demographic and genetic datasets. SCN-resistant and SCN-susceptible soybean cultivars with a combination of soybean aphids (biotype 1) and SCN (HG type 0) in a randomized complete block design (RCBD) with six blocks were used to evaluate the three-way interactions in a greenhouse setup. Treatments receiving SCN were infested at planting with 2000 nematode eggs, and the treatments with soybean aphids were infested at second trifoliate growth stage (V2) with 15 soybean aphids. The whole roots were sampled from plants at 5 and 30 days post SBA infestation for RNA sequencing using Illumina Hiseq. 3000. The data comprises of 47 libraries that are useful for further analyses of important genes, which are involved in interaction effects of SBA and SCN on soybean.

## Abstract (summary)

**Table:** 

Design Type(s)	transcription profiling design • randomized complete block design • strain comparison design • stimulus or stress design
Measurement Type(s)	transcription profiling assay
Technology Type(s)	RNA sequencing
Factor Type(s)	cultivar • temporal_interval • experimental condition • biological replicate
Sample Characteristic(s)	Glycine max • root • cropland biome

Machine-accessible metadata file describing the reported data (ISA-Tab format)

## Background & Summary

Soybean [*Glycine max* (L.) Merr.], considered as the source of high-quality sugar, protein, and oil, is one of the most important crops worldwide^1^. Soybean aphid (SBA), *Aphis glycines* Matsumura (Hemiptera: Aphididae) and soybean cyst nematode (SCN), *Heterodera glycines* Ichinohe (Tylenchida: Heteroderidae) are the two most economically important pests of soybean in the Midwestern United States^2,3^. Soybean aphid, an aboveground herbivore (pest), feeds on phloem sap whereas SCN, a belowground pest, infests the soybean roots. These infestations can co-occur and amplify further reduction in soybean yield^4,5^. In the United States, annual economic losses due to the SBA and SCN have been estimated to be approximately $4 billion and $1.3 billion, respectively^6–8^. To counteract these devastating pests, farmers rely on various management strategies that include host plant resistance and chemical measures^9–11^. For SBA, dependency on the use of chemical management has resulted in pyrethroid resistance in SBA populations in Iowa, Minnesota, North Dakota and South Dakota as well as the impacts on non-target beneficial organisms^12,13^. In addition, the long-term use of SCN resistance has resulted in SCN populations that are capable of overcoming the resistance genes (i.e., HG types)^14^. Although host plant resistance has not been implemented on a large scale for SBA management, multiple virulent SBA biotypes have been discovered in the U.S. Virulent SBA biotypes and SCN races threaten the sustainability of host plant resistance for these two pests^14–17^. Thus, genetic data generated from greenhouse experiments on the effects of SBA and SCN on soybean cultivars are of tremendous importance for unraveling resistance genes and regulatory networks that can potentially be used for developing durable resistance in soybean to both pests.

Although above- and below- ground herbivores are spatially segregated, they both share the host plant through systemic tissues and are able to influence each other^18^. Previously, the influence of SCN on soybean aphid infestation or *vice versa* has been studied on soybean using demographic datasets^4,5,19–21^. McCarville, *et al*.^4^ conducted experiments on various soybean cultivars [SCN susceptible (DK 28–52, IA 3018, IA 3041) and SCN resistant (DK 27–52, AG 2821 V, IA 3028)] to understand the effect of SBA, SCN, and fungus *Cadophora gregata* (Allington & Chamberlain) Harrington & McNew on soybean. Their study showed 5.24 times increase in SCN reproduction in the presence of soybean aphid and the fungus. In contrast, the aphid population decreased by 26.4% in the presence of SCN and *C*. *gregata* and the aphid exposure reduced by 19.8% in SCN resistant cultivars. Later, McCarville, *et al*.^5^ demonstrated the relationship between the aboveground feeding of soybean aphid and belowground reproduction of SCN in the SCN resistant Dekalb 27–52 (PI 88788 derived) cultivar, and SCN susceptible Kenwood 94 cultivar. In 30 days, both SCN eggs and the number of females increased by 33% in SCN-resistant cultivar and reduced by 50% in the SCN-susceptible cultivar. In 60 days, the number of SCN eggs and female count remained unaffected in the resistant cultivar but decreased in the susceptible cultivar. The authors concluded that soybean aphid feeding improved the quality of soybean as a host for SCN but this result was varied significantly with the cultivar and length of the experiment. Apart from these demographic studies, molecular characterization of SBA-SCN-soybean interaction has not been reported previously.

**Fig. 1:**
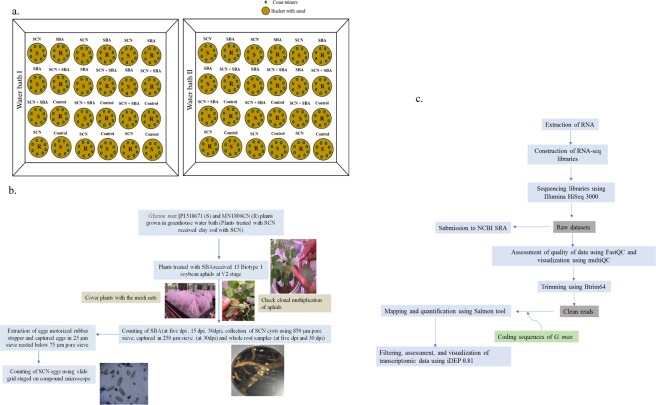
An overview of greenhouse experiments and transcriptomic data analysis pipeline. (**a**) A randomized complete block design (RCBD) using two water baths (Water bath I and Water bath II), (**b**) A flow chart representing experimental methods used for soybean cyst nematode and soybean aphid interaction using two cultivars of soybean, and (**c**) A flow chart showing RNA-seq data analysis pipeline.

RNA-Sequencing (RNA-Seq) has been a standard tool for studying qualitative and quantitative gene expression assays that provide information on transcript abundance with their variation^22,23^. The major objective of this study was to evaluate differential gene expression of soybean plants that are infested with SCN in the presence or absence of SBA. To achieve the objective, we conducted experiments on two genotypes of *G*. *max* [*H*. *glycines* susceptible Williams 82 (PI518671), and *H*. *glycines* resistant MN1806CN] that were infested with biotype 1 SBA and HG Type 0 SCN for RNA-sequencing. More than 1.1 billion reads (61.4 GB) of transcriptomic data were obtained from 47 samples derived from the experiment using whole roots of *G*. *max*. An overview of the experimental design, methods and transcriptome analysis pipeline is shown in Fig. 1a–c, respectively. A comprehensive understanding of these transcriptome data will enhance our understanding of interactions among soybean, SBA, and SCN at the molecular level. The rapid advancement of bioinformatics tools is facilitating the search of candidate genes and their function that might play a crucial role in various pathways for host resistance against both herbivores.

## Methods

### Plant material, soybean aphid, and SCN

Two cultivars of soybean– Williams 82 (PI518671) and MN1806CN were used in this experiment. Williams 82 is susceptible to both HG Type 0 (race 3) of the SCN and SBA. MN1806CN is resistant to HG Type 0 (race 3) of the SCN but susceptible to SBA. Soybean aphid biotype 1 populations were originally obtained from the Ohio State University and were reared on susceptible cultivar LD12-15838R at South Dakota State University. This biotype is defined by an avirulent response to all known SBA resistance (*Rag*) genes and was first identified in Illinois^24^. The SCN population used was HG type 0, which is defined by having less than 10% reproduction documented by studies of SCN resistance and is avirulent to all SCN resistance genes in soybean.

### Experimental design and sample collection

A greenhouse experiment was designed using a randomized complete block design (RCBD) with eight treatments (four treatments per cultivars) with eight experimental units (plants) in six blocks. The treatments were factors of soybean genotype, SBA infestation, and SCN infestation. For examples, each of the soybean genotypes received one of the following combinations: SCN:no SBA, no SCN:SBA, SCN:SBA, or no SBA:no SCN (control).

For this experiment, the soil-sand mixture was prepared by adding construction sand and clay soil including SCN infested clay soil in the ratio of 3:1. The 125 cc of the mixture was distributed in cone-tainers (diameter of 3.8 cm, a depth of 21 cm and a volume of 164 cc; Greenhouse Megastore, USA). For SCN included treatments, each cone-tainer received approximately 2,000 SCN eggs. The cone-tainers with three soybean seeds were arranged in a 2.0 U.S. gallon (7.57 liter) plastic buckets (Leaktite, USA) filled with construction sand (Quikrete, GA). These buckets were kept in a water bath for maintaining soil temperature between 26.7 °C and 28.9 °C to ensure the reproduction of SCN (i.e. ~30 days)^5^. The temperature of the water bathes were regularly monitored using thermometers. The plants were grown under 16:8 (L:D) in a greenhouse with a temperature of 28 °C and 45% relative humidity. The plants were thinned down to one plant per cone-tainer upon reaching the second vegetative growth stage (V2). The V2-staged plants with the SBA included treatments were infested with 15 mixed age (i.e., fourth instar nymphs and adults) biotype 1 SBA using a 000 fine tip paintbrush (Winsor & Newton, England). The SBA were applied on the abaxial surface of the first trifoliate of V2-staged plants. All plants in each bucket were covered with a large no-see-um mesh net (Quest Outfitters, Sarasota, FL) to prevent inter-bucket movement of aphids. After SBA infestation, soybean plants were regularly checked to confirm the successful establishment of soybean aphids. Soybean aphid populations were counted at 5, 15, and 30 days post infestation (dpi). For SBA only treatment, the populations on the two soybean varieties were not significantly different, indicating that both lines were susceptible to SBA. SCN eggs were sampled at 30 dpi. The whole roots were collected on 5 and 30 dpi by snap freezing in liquid Nitrogen and stored at −80 °C for further analysis. The 5 dpi and 30 dpi root samples treated with each treatment were collected from Water bath I and Water bath II, respectively, representing each plant from three blocks (three biological replicates). The SCN soil and SCN infested roots were used for SCN cysts collection (except root samples collected for transcriptomic study) and the soil was examined for SCN counts.

### RNA extraction, library construction, and RNA sequencing

RNA was extracted from all samples representing three biological replicates of each treatment that constituted 24 samples collected at 5 and 30 dpi each. As the major foci of the project were to determine whether the gene expression differed between SCN resistant and SCN susceptible soybeans, and to evaluate the gene expression of soybeans that were dual infested with SCN and SBA, we selected two timepoints (5 and 30 dpi). We selected 30 dpi to observe gene expression of treatment effects on a single generation of SCN reproduction keeping 5 dpi as a reference in the presence or absence of SBA. Frozen root samples from each treatment were grounded in liquid nitrogen with a mortar and pestle to a fine powder followed by total RNA extraction using PureLink RNA mini kit (Invitrogen, USA). RNA samples were treated with TURBO^TM^ DNase (Invitrogen, USA) to remove any DNA contamination following the manufacturer’s instructions. Assessment of the isolated RNA integrity was performed by 1% agarose gel electrophoresis, and RNA concentration was measured by Nanodrop 2000 (Thermo Fisher Scientific, USA). The cDNA libraries were constructed using NEBNext Ultra II RNA library 96 single index prep kit and sequenced using Illumina HiSeq. 3000 (single read end utilizing 100 bases read length) at Iowa State University Sequencing Facilities.

### Pre-processing of sequencing data

Quality control of reads was assessed using FastQC program (version 0.11.3) (https://www.bioinformatics.babraham.ac.uk/projects/fastqc/)^25^. The FastQC results were visualized using MultiQC v1.3^26^, and low quality bases (QC value < 20; 5-bp window size) were removed by trimming in the program Btrim64 (version 0.2.0)^27^. High-quality single-end reads were mapped against the primary coding sequences of *G*. *max*. The coding sequences (*Gmax: Gmax_275_Wm82*.*a2*.*v1*.*transcript_primaryTranscriptOnly*.*fa*.*gz*) were obtained from the Phytozome database and aligned using Salmon ver.0.9.1^28^ accessed from Bioconda^29^. Downstream analyses of the quantified transcript reads were performed using integrated Differential Expression and Pathway analysis (iDEP 0.81, R/Bioconductor packages)^30^. The quantified transcript reads were filtered with 0.5 counts per million (CPM) in at least one sample and transformed using regularized log (rlog), which is implemented in the DESeq. 2^31^ package.

## Data Records

All sequence reads were deposited in the National Center for Biotechnology Information (NCBI) Sequence Read Archive (accession SRR8427366-SRR8427408) under Bioproject PRJNA514200 (Project ID: SRP178193)^33^ (Table 1). The raw transcript abundance counts for all the samples was deposited at the Gene Expression Omnibus (GEO) database, GSE125103^34^. The transformed transcript abundance counts, hierarchical clustering, correlation matrices, and clusters are available in figshare at: 10.6084/m9.figshare.7755152.v3^32^.

**Table 1:** Statistics of the transcriptomic data using RNA-seq pipeline used in this study.

Sample	Number of raw reads	GC %	Read Length	Trimmed reads	Percentage of clean reads	Mapped Reads	Percentage of mapped reads	Number of Uniquely mapped reads	Percent uniquely mapped	Accession
PI518671_treatment_SCN_30d_R1	29,875,777	44	99	29,868,305	99.97%	26,306,640	88.1	24,916,413	83.4	SRR8427366
PI518671_treatment_SCN_30d_R2	20,569,129	45	99	20,564,513	99.98%	18,327,957	89.1	17,356,148	84.4	SRR8427367
PI518671_treatment_SCN_30d_R3	23,663,582	44	99	23,657,909	99.98%	20,899,976	88.3	19,646,683	83.0	SRR8427368
PI518671_treatment_Aphid_30d_R1	24,553,476	45	99	24,546,368	99.97%	21,032,002	85.7	19,429,157	79.2	SRR8427369
PI518671_treatment_Aphid_30d_R2	25,372,180	45	99	25,364,647	99.97%	22,011,320	86.8	19,706,012	77.7	SRR8427362
PI518671_treatment_Aphid_30d_R3	37,691,731	44	99	37,682,590	99.98%	31,646,750	84.0	29,865,320	79.3	SRR8427363
PI518671_treatment_SCNAphid_30d_R1	23,727,017	45	99	23,721,761	99.98%	21,457,335	90.5	20,276,187	85.5	SRR8427364
PI518671_treatment_SCNAphid_30d_R2	22,378,982	44	99	22,373,777	99.98%	19,622,486	87.7	18,602,604	83.1	SRR8427365
PI518671_treatment_SCNAphid_30d_R3	27,673,846	44	99	27,668,291	99.98%	23,304,305	84.2	22,080,120	79.8	SRR8427370
MN1806CN_treatment_SCN_30d_R1	25,200,882	43	99	25,192,664	99.97%	18,589,872	73.8	17,402,401	69.1	SRR8427371
MN1806CN_treatment_SCN_30d_R2	22,192,100	43	99	22,186,459	99.97%	18,350,922	82.7	17,417,979	78.5	SRR8427383
MN1806CN_treatment_SCN_30d_R3	20,653,286	43	99	20,648,111	99.97%	15,975,636	77.4	15,083,771	73.1	SRR8427384
MN1806CN_treatment_Aphid_30d_R1	20,903,446	44	99	20,896,290	99.97%	17,025,027	81.5	15,982,207	76.5	SRR8427385
MN1806CN_treatment_Aphid_30d_R2	21,708,115	44	99	21,701,712	99.97%	16,458,081	75.8	15,472,937	71.3	SRR8427386
MN1806CN_treatment_Aphid_30d_R3	26,617,069	44	99	26,610,582	99.98%	22,222,510	83.5	21,021,087	79.0	SRR8427387
MN1806CN_treatment_SCNAphid_30d_R1	19,498,275	43	99	19,491,491	99.97%	15,139,964	77.7	14,203,387	72.9	SRR8427388
MN1806CN_treatment_SCNAphid_30d_R2	27,765,044	44	99	27,759,095	99.98%	22,021,174	79.3	20,747,251	74.7	SRR8427389
MN1806CN_treatment_SCNAphid_30d_R3	43,325,617	44	99	43,312,161	99.97%	33,076,203	76.4	29,935,328	69.1	SRR8427390
MN1806CN_treatment_control_30d_R1	24,104,763	45	99	24,099,789	99.98%	18,112,259	75.2	17,132,109	71.1	SRR8427391
MN1806CN_treatment_control_30d_R2	32,183,362	44	99	32,174,938	99.97%	26,274,456	81.7	24,162,028	75.1	SRR8427392
PI518671_treatment_control_30d_R1	20,522,473	44	99	20,518,044	99.98%	17,937,163	87.4	17,022,590	83.0	SRR8427405
PI518671_treatment_control_30d_R2	28,600,503	44	99	28,593,731	99.98%	25,409,842	88.9	24,045,140	84.1	SRR8427404
PI518671_treatment_control_30d_R3	20,577,190	44	99	20,570,977	99.97%	17,574,516	85.4	16,585,012	80.6	SRR8427407
PI518671_treatment_SCN_5d_R1	20,389,378	44	99	20,383,629	99.97%	17,826,706	87.5	16,736,123	82.1	SRR8427406
PI518671_treatment_SCN_5d_R2	10,518,888	44	99	10,516,365	99.98%	9,444,170	89.8	8,950,048	85.1	SRR8427401
PI518671_treatment_SCN_5d_R3	21,303,947	44	99	21,298,111	99.97%	18,909,955	88.8	17,897,118	84.0	SRR8427400
PI518671_treatment_Aphid_5d_R1	20,262,293	45	99	20,256,610	99.97%	18,157,064	89.6	16,851,551	83.2	SRR8427403
PI518671_treatment_Aphid_5d_R2	51,680,716	44	99	51,666,055	99.97%	45,293,720	87.7	42,794,964	82.8	SRR8427402
PI518671_treatment_Aphid_5d_R3	20,328,355	44	99	20,322,387	99.97%	18,171,819	89.4	17,083,986	84.1	SRR8427399
PI518671_treatment_SCNAphid_5d_R1	21,569,888	44	99	21,563,432	99.97%	18,502,664	85.8	17,044,428	79.0	SRR8427398
PI518671_treatment_SCNAphid_5d_R2	57,520,568	44	99	57,503,170	99.97%	47,902,174	83.3	45,268,224	78.7	SRR8427381
PI518671_treatment_SCNAphid_5d_R3	16,889,301	45	99	16,883,954	99.97%	14,700,125	87.1	13,744,624	81.4	SRR8427382
MN1806CN_treatment_SCN_5d_R1	25,443,012	44	99	25,435,147	99.97%	21,929,527	86.2	20,483,059	80.5	SRR8427379
MN1806CN_treatment_SCN_5d_R2	20,043,049	45	99	20,037,212	99.97%	17,551,266	87.6	16,336,263	81.5	SRR8427380
MN1806CN_treatment_SCN_5d_R3	9,847,269	45	99	9,844,767	99.97%	8,472,717	86.1	7,992,925	81.2	SRR8427377
MN1806CN_treatment_Aphid_5d_R1	20,503,738	45	99	20,497,489	99.97%	16,815,160	82.0	15,666,380	76.4	SRR8427378
MN1806CN_treatment_Aphid_5d_R2	14,359,303	45	99	14,355,678	99.97%	12,268,563	85.5	11,559,112	80.5	SRR8427375
MN1806CN_treatment_Aphid_5d_R3	19,094,540	45	99	19,088,178	99.97%	16,590,158	86.9	15,245,807	79.9	SRR8427376
MN1806CN_treatment_SCNAphid_5d_R1	20,636,498	44	99	20,630,026	99.97%	16,806,607	81.5	15,865,622	76.9	SRR8427373
MN1806CN_treatment_SCNAphid_5d_R2	22,488,050	44	99	22,482,625	99.98%	19,286,899	85.8	18,060,389	80.3	SRR8427374
MN1806CN_treatment_SCNAphid_5d_R3	22,033,213	45	99	22,028,303	99.98%	16,862,396	76.5	15,964,103	72.5	SRR8427408
MN1806CN_treatment_control_5d_R1	18,937,367	46	99	18,932,017	99.97%	14,805,819	78.2	12,707,453	67.1	SRR8427396
MN1806CN_treatment_control_5d_R2	26,710,585	43	99	26,702,238	99.97%	20,226,195	75.7	18,092,239	67.8	SRR8427394
MN1806CN_treatment_control_5d_R3	21,327,385	46	99	21,320,799	99.97%	16,776,843	78.7	14,820,338	69.5	SRR8427372
PI518671_treatment_control_5d_R1	17,242,793	45	99	17,239,066	99.98%	16,044,618	93.1	14,976,834	86.9	SRR8427397
PI518671_treatment_control_5d_R2	22,062,929	46	99	22,055,685	99.97%	20,094,996	91.1	17,347,038	78.7	SRR8427395
PI518671_treatment_control_5d_R3	21,220,300	44	99	21,213,623	99.97%	19,994,447	94.3	18,592,042	87.6	SRR8427393

## Technical Validation

### Quality control

**Fig. 2:**
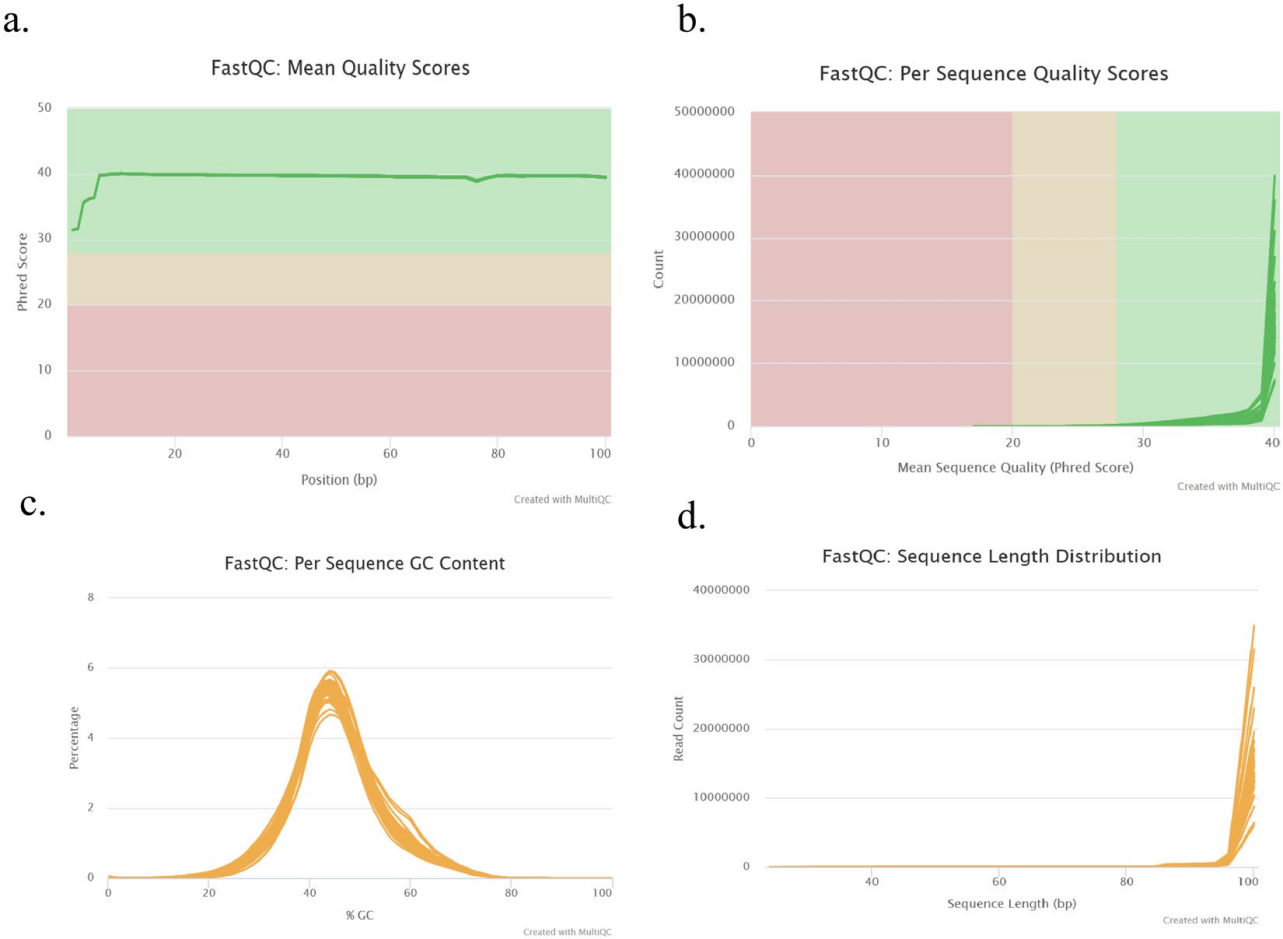
Quality metrics of *G*. *max* sequencing data. (**a**) Mean quality scores per position, (**b**) Per sequence quality scores, (**c**) GC content distribution, and (**d**) Read length distribution.

Forty-eight RNA libraries were prepared and sequenced with the sequencing depth ranging from 9,847,269 to 57,520,568 reads (see Table 1). Sequencing of one of the replicates of control resistant cultivar (MN1806CN collected at 30 dpi) came in error. Therefore, total reads of more than 1.1 billion from 47 libraries were subjected to FastQC analysis, which helped determine the data quality using various quality metrics such as mean quality scores (Fig. 2a), per sequence quality scores (Fig. 2b), per sequence GC content (Fig. 2c), and sequence length distribution (Fig. 2d). Phred quality scores per-base for all samples were higher than 30 and GC content ranged from 43 to 45%, following a normal distribution. After trimming, more than 99% of the reads were retained as clean and good quality reads. Upon mapping these reads, a high mapping rate of 73.8% to 94.3% was obtained. Among these, 67.1% to 87.6% reads were uniquely mapped.

### Assessment of transcriptomic data

**Fig. 3:**
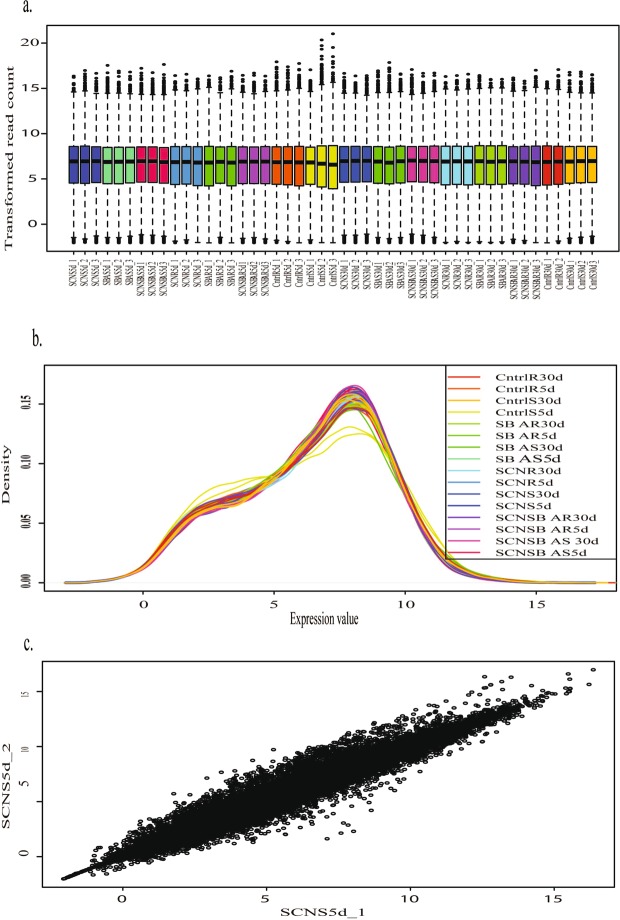
Pre-processing of transcriptomic data. (**a**) Distribution of transformed data, (**b**) Density plot of transformed data, and (**c**) Scatter plot of the first two samples (SCNS5d_1 vs SCNS5d_2).

**Fig. 4:**
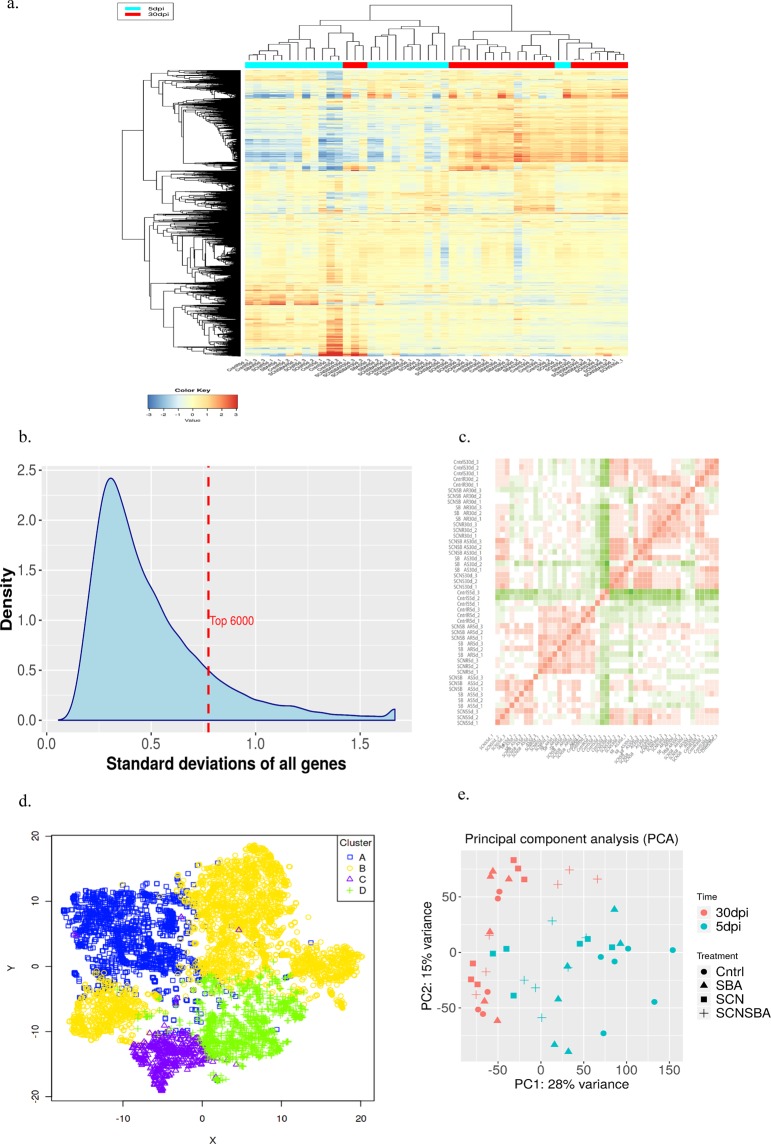
Assessment of transcriptomic data. (**a**) Heatmap of top 6,000 variable genes, (**b**) Gene SD distribution, (**c**) Correlation matrix, (**d**) Visualization of top 6,000 genes shown in the t-SNE map, and (**e**) A principal components analysis (PCA) plot.

The 43,122 genes passed the filter upon filtering with 0.5 CPM in at least one sample. To reduce the mean dependent variance, the quantified transcript reads were transformed as shown in Fig. 3a–c and available in Figshare^32^ (the transformed transcript abundance count for all the samples). The transformed data were subjected to hierarchical clustering and principal component analysis (PCA) followed by visualization using t-SNE map^35^ in order to assess the global transcriptomic data. The hierarchical clustering of top 6000 variable genes based on two time points (5 dpi and 30 dpi) showed distinct clustering except for some samples [Fig. 4a; Figshare^32^ (the hierarchical clustering of top 6,000 variable genes)]. Figure 4b represents the standard deviation (SD) distribution of the top variable 6,000 genes. Figure 4c represents the Pearson’s correlation between the samples using the top 75% genes and available in Figshare^32^ (the correlation between the samples using the top 75% genes). The t-SNE map revealed four clusters (A, B, C, and D) for 6,000 variable genes [Fig. 4d; Figshare^32^ (the four clusters for 6,000 variable genes)]. Regarding the PCA, PC1 is correlated with time (*P* = 1.16e-06) with 28% variance, and PC2 is correlated with Treatment (*P* = 2.02e-08) with 15% variance (Fig. 4e).

## Usage Notes

This data represents the first publicly available transcriptomic data for soybean roots from the three-way interaction among *G*. *max*, *H*. *glycines*, and *A*. *glycines*. The raw compressed fastq files (fastq.gz) were submitted to the National Center for Biotechnology Information (NCBI) and are available with accession numbers (SRR8427366-SRR8427408; http://identifiers.org/ncbi/insdc.sra:SRP178193)^33^. The data could be retrieved using fastq-dump tool SRA toolkit (https://www.ncbi.nlm.nih.gov/sra). There are various tools such as Trimmomatic^36^, cutadapt^37^, Fastq_clean^38^ that could be used for trimming purpose. Apart from the Salmon tool for the alignment and quantification of reads, other tools such as STAR aligner (https://github.com/alexdobin/STAR), Bowtie^39^, HISAT2^40^, TopHat2^41^, Cufflinks with HTSeq can be employed, which requires reference genome of *G*. *max* and annotation file in *gff3* format. For differential gene expression analysis, EdgeR^42^ and limma^43^ could be used instead of DEseq. 2^31^. Apart from the standalone tools like iDEP^30^, Galaxy (https://usegalaxy.org), CyVerse (http://www.cyverse.org), MeV (http://mev.tm4.org)^44^, and integrated RNA-seq interpretation system for gene expression data analysis tool (http://bmbl.sdstate.edu/IRIS/)^45^ could also be used for both analysis and visualization of RNA-seq data.

## ISA-Tab metadata file

Download metadata file

## Data Availability

Codes used for RNA-seq data processing in the current study are available as supplementary material in Figshare at: 10.6084/m9.figshare.7755152.v3^32^ (Codes used for RNA-seq data processing).
